# Interaction and Joint Effects of Inflammation and Triglyceride–Glucose (TyG) Index on Hyperuricemia Risk and the Mediating Role of TyG: A Cross‐Sectional Study

**DOI:** 10.1002/edm2.70205

**Published:** 2026-03-30

**Authors:** Xueran Huang, Pengfei Chen, Yongfen Gao, Zengnan Mo, Haiying Zhang, Rui Lin

**Affiliations:** ^1^ School of Public Health Guangxi Medical University Nanning China; ^2^ Center for Genomic and Personalized Medicine, Guangxi Key Laboratory for Genomic and Personalized Medicine University Engineering Research Center of Digital Medicine and Healthcare, Guangxi, Guangxi Medical University Nanning China; ^3^ Guangxi Colleges and Universities Key Laboratory of Prevention and Control of Highly Prevalent Diseases Guangxi Medical University Nanning China

**Keywords:** hyperuricemia, inflammation score, interaction effect, mediation effect, triglyceride–glucose index

## Abstract

**Background:**

The chronic inflammation and insulin resistance (IR) are implicated in hyperuricemia (HUA) pathogenesis, while their interplay remains inadequately characterised. This study aimed to investigate their independent, interactive, and combined effects on HUA risk and serum uric acid (UA) levels, and to quantify the mediating effect of IR in the Southern Chinese population.

**Methods:**

A cross‐sectional study was conducted among 5307 Chinese adults. The triglyceride–glucose (TyG) index served as an alternative marker for IR, and a modified inflammation (mINFLA) score, comprising white blood cell (WBC) count, platelet (PLT) count, and the neutrophil‐to‐lymphocyte ratio (NLR), was used to assess systemic low‐grade inflammation. The multivariable logistic and linear regression models were employed to explore the associations of the TyG index and mINFLA score with HUA risk and UA levels, and the interaction effects and mediating role of the TyG index were further assessed. Subgroup analyses were stratified by age, sex, and disease status.

**Results:**

Among 5307 Chinese adults, a higher TyG index and a higher mINFLA score were significantly associated with an increased HUA risk and elevated UA levels, demonstrating significant dose–response relationships (*p* for trend < 0.001). A significant interaction was observed between the TyG index and mINFLA score in relation to HUA risk and UA levels. Participants exhibiting both high TyG index and mINFLA score had the highest HUA risk and UA levels. Notably, mediation analysis revealed that the TyG index significantly explained 15.4% of the association between the mINFLA score and HUA risk, and 14.5% of the association with UA levels.

**Conclusion:**

Chronic inflammation is an independent risk factor for hyperuricemia, with insulin resistance serving as a key mediating factor. Furthermore, these two factors exhibit a significant joint effect on hyperuricemia risk. These findings underscore the importance of simultaneously considering both insulin resistance and chronic inflammation in hyperuricemia prevention and management.

## Graphical abstract

Among 5307 southern Chinese adults, higher TyG index and mINFLA score were associated with increased HUA risk and serum UA levels (*p* trend < 0.001). A significant interaction was observed between these two indicators. Notably, the TyG index mediated 15.4% of the mINFLA‐HUA association and 14.5% of the mINFLA‐UA association.

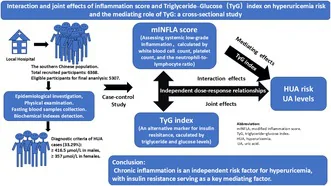

## 1 Introduction

Hyperuricemia (HUA) is a chronic metabolic disorder characterised by elevated serum uric acid (UA) levels. Poorly controlled hyperuricemia may progress to serious complications such as cardiovascular disease, chronic kidney disease, and diabetes [1]. Specifically, this burden exhibits regional disparities, as evidenced by a systematic review indicating a notably higher prevalence in Southern China compared to other regions (prevalence 25.5% vs. 15.4%) [2], highlighting the need for research in Southern Chinese populations. It has been demonstrated that UA possesses a multifaceted role including antioxidant, pro‐oxidative, pro‐inflammatory, and immune effects, whose equilibrium is involved in crosstalk among various epithelial tissues and cell types [3]. While HUA, resulting from an imbalance between UA production and excretion, is the consequence of complex interactions among various risk factors [3]. However, it is not well established whether and how insulin resistance and chronic low‐grade inflammation interplay on HUA risk, especially in the Southern Chinese population.

Insulin resistance (IR), a key pathophysiological factor in HUA, reduced uric acid clearance rate and subsequently promoted serum UA elevation at kidney level [4]. However, the relationship between IR and HUA remains debated. A Mendelian randomization study demonstrated that genetically proxied reduction in IR leads to lower acid levels [5]. Conversely, elevated uric acid has been shown to induce or exacerbate IR through mechanisms such as impaired endothelial nitric oxide synthesis [6] and activation of the NLRP3 inflammasome [7]. The triglyceride–glucose (TyG) index, calculated from fasting triglyceride and glucose levels, has emerged as a reliable and accessible alternative marker for IR. It offers a practical advantage over the gold standard of hyperinsulinemic‐euglycemic clamp and is widely employed in epidemiological studies of cardiometabolic diseases [8]. Recent retrospective and nationwide cohort studies further identify the TyG index as an independent predictor of HUA [9, 10]. Beyond IR, systemic low‐grade inflammation is increasingly recognised as a critical player in HUA pathogenesis. Individual inflammatory markers, including C‐reactive protein (CRP) [11], white blood cell (WBC) count [12], platelet (PLT) count [13], and the neutrophil‐to‐lymphocyte ratio (NLR) [14], have each been independently associated with HUA. However, the inflammatory response is a complex integrated process, and a composite measure may provide a more holistic assessment of an individual's inflammatory status. The INFLA‐score, a validated composite index incorporating CRP, WBC, PLT, and NLR, has been applied to investigate the role of chronic low‐grade inflammation in conditions like obesity, type 2 diabetes, and other cardiovascular diseases [15, 16, 17, 18]. In this study, we employed a modified INFLA (mINFLA) score, comprising WBC, PLT, and NLR, to circumvent the routine unavailability of CRP in the health physical examination.

While both the IR and systemic inflammation are independently linked to HUA, their interplay and potential mediating mechanism are yet to be elucidated. Therefore, this study aims to comprehensively investigate the independent, interactive, and joint effects of the TyG index and mINFLA score on the risk of HUA in a population from Southern China. The findings are expected to provide deeper insights into the underlying mechanisms and inform integrated strategies for the prevention and management of HUA.

## 2 Methods

### 2.1 Study Participants

In this cross‐sectional study, a total of 6368 subjects undergoing health physical examination were recruited from Southern China at the healthcare centers in Nanning, Yulin, and Guilin City, from February 2018 to December 2023. As shown in Figure S1, subjects with cancer, mental illness, severe neurological disorders, severe organic diseases, acute infection, and less than 18 years old were excluded, and 994 individuals were excluded for incomplete questionnaire (*n* = 50) or missing biochemical indicators including serum UA (*n* = 191), lymphocyte count (LYM), neutrophil count (NEU) (*n* = 732), or fasting blood glucose (FBG) (*n* = 21), leaving a total of 5307 eligible subjects for the final analysis.

### 2.2 Data Collection

Face‐to‐face interviews were conducted by trained investigators using a valid questionnaire to collect information on participants' demographic characteristics, lifestyle factors, medical history, medication use, and surgical history. The physical examination and the fasting blood samples collection were conducted by the local nurses. The serum FBG, triglyceride (TG), UA, and other biochemical indexes were detected by chemiluminescence technology analysis.

### 2.3 Definition of mINFLA Score and TyG Index

The INFLA score conventionally comprises four inflammation indices [18]: C‐reactive protein (CRP), white blood cell (WBC) count, platelet (PLT) count, and the neutrophil‐to‐lymphocyte ratio (NLR). As CRP is not routinely measured in standard health examinations, we employed a modified INFLA (mINFLA) score comprising only WBC count, PLT count, and NLR to assess the low‐grade inflammation. Each component of the modified mINFLA score is divided into deciles. The 7th to 10th deciles are assigned values of +1 to +4, while the 1st to 4th deciles are assigned values of −4 to −1, and 5th to 6th deciles are assigned 0 value. Then all the values are summed; subsequently, the final mINFLA score ranges from −12 to +12, with a higher score indicating a more severe low‐grade inflammation. Higher scores reflect higher levels of low‐grade inflammation.

The TyG index was calculated by the following formula [8]: TyG = Ln [fasting triglyceride (mg/dL) × fasting glucose (mg/dL)/2].

### 2.4 Definition of DVS

Dietary habits were assessed using a short food frequency questionnaire that inquired about the weekly consumption frequency over the previous 12 months. The dietary variety score (DVS) was calculated based on seven food items, adapted from instruments used in Taiwan, China [19] and Japan [20].

### 2.5 Definition of Disease Status

Hyperuricemia (HUA) was defined as a serum uric acid (UA) level ≥ 416.5 μmol/L in males and ≥ 357 μmol/L in females [21]. Obesity [22] was defined as a body mass index (BMI) ≥ 28 kg/m^2^. Hypertension (HBP) [23] was diagnosed if any of the following criteria were met: systolic blood pressure (SBP) ≥ 140 mmHg, diastolic blood pressure (DBP) ≥ 90 mmHg, self‐reported physician diagnosis, or current use of antihypertensive medication. Type 2 diabetes mellitus (T2DM) [24] was diagnosed based on any of the following: fasting blood glucose (FBG) ≥ 7.0 mmol/L, 2‐h postprandial blood glucose during an oral glucose tolerance test (OGTT) ≥ 11.1 mmol/L, glycated haemoglobin (HbA1c) ≥ 6.5%, self‐reported physician diagnosis, or current use of antidiabetic medication. Chronic kidney disease (CKD) [25] was defined as an estimated glomerular filtration rate (eGFR) < 90 mL/min/1.73 m^2^.

### 2.6 Statistical Analysis

Differences between two groups were assessed using *t*‐tests or rank sum tests for continuous variables, and chi‐square tests for categorical variables. Continuous variables were presented as mean ± standard deviation (SD) or median with interquartile range (IQR 5th–95th percentiles). Categorical variables were summarised as frequency percentages. The mINFLA score was converted using the interquartile range (IQR) for subsequent analysis [26]. The potential nonlinear associations of mINFLA score and TyG index with HUA risk were examined using restricted cubic splines (RCS). Since RCS analyses showed a linear relationship between mINFLA score and HUA risk while a nonlinear relationship between TyG index and HUA risk, the median of mINFLA score (median = 0, Figure S2) and the inflection point of TyG index (inflection point = 8.47, Figure S3) were used to categorise the participants into High and Low groups, respectively. Participants were then classified into four groups: low mINFLA & low TyG, low mINFLA & high TyG, high mINFLA & low TyG, and high mINFLA & high TyG. Multivariable logistic regression was applied to evaluate the independent and combined effects of their effects on the odds of HUA. Multivariable linear regression was used to assess their independent and combined effects on serum UA levels. Finally, we performed mediation analysis via the mediation package in R software (version 4.5.1) [27]. All analyses were performed using Stata (StataCorp LP, USA, version 12) and R (version 4.5.1), with a two‐sided *p*‐value < 0.05 considered statistically significant.

### 2.7 Ethics Statement

This study was approved by the Ethics Committee of Guangxi Medical University (Ethics Approval No. 158 (2017) and No. 0015 (2022)) and in accordance with the principles stipulated in the Helsinki Declaration. The written informed consent was signed by all the participants before data collection.

## 3 Results

### 3.1 Characteristics of Participants

Among the 5307 study subjects, the median age was 47 years old, and 33.29% were HUA cases (*n* = 1767). The cases with HUA demonstrated significant differences across multiple clinical and lifestyle factors compared to the controls (Table 1). Specifically, the case group had a significantly higher prevalence of comorbidities (e.g., type 2 diabetes, hypertension, chronic kidney disease), adverse metabolic parameters (e.g., elevated FBG, lipids, CREA, UA, mINFLA score and TyG index), and lower HDL‐C and eGFR. Significant differences were also noted in sociodemographic and behavioural characteristics (e.g., smoking, alcohol, tea, and sports).

**TABLE 1:** The basic characteristics of the study subjects.

Characteristics	Total (*n* = 5307)	Control (*n* = 3540)	Case (*n* = 1767)	*p*
Age, years	47 (25, 75)	47 (25, 75)	47 (25, 74)	0.321
Male	3423 (64.5)	1974 (55.76)	1449 (82)	< 0.001***
BMI, kg/m^2^	23.34 (18.2, 29.17)	22.58 (17.9, 28.16)	24.86 (19.82, 30.86)	< 0.001***
DVS	15 (9, 20)	15 (8, 20)	15 (10, 20)	< 0.001***
Region				
Nanning	2526 (47.6)	1805 (50.99)	721 (40.8)	< 0.001***
Yulin	2096 (39.5)	1162 (32.82)	934 (52.86)	
Guilin	685 (12.91)	573 (16.19)	112 (6.34)	
Ethnicity				
Han ethnicity	4664 (87.88)	3086 (87.18)	1578 (89.3)	< 0.001***
Zhuang ethnicity	414 (7.8)	267 (7.54)	147 (8.32)	
Others	229 (4.32)	187 (5.28)	42 (2.38)	
Education				
Below primary school	1050 (19.79)	790 (22.32)	260 (14.71)	< 0.001***
Middle school	940 (17.71)	589 (16.64)	351 (19.86)	
Above college	3317 (62.5)	2161 (61.05)	1156 (65.42)	
Occupation				
Office staff	1663 (31.34)	1107 (31.27)	556 (31.47)	0.977
Professionals	3167 (59.68)	2113 (59.69)	1054 (59.65)	
Other	477 (8.99)	320 (9.04)	157 (8.89)	
Married	4068 (76.65)	2663 (75.23)	1405 (79.51)	< 0.01**
Annual income (RMB)				
≤ 50,000	2000 (37.69)	1424 (40.23)	576 (32.6)	< 0.001***
50,000–100,000	1876 (35.35)	1220 (34.46)	656 (37.13)	
≥ 100,000	1431 (26.96)	896 (25.31)	535 (30.28)	
Smoking				
Current	1127 (21.24)	679 (19.18)	448 (25.35)	< 0.001***
Ever	422 (7.95)	257 (7.26)	165 (9.34)	
Never	3758 (70.81)	2604 (73.56)	1154 (65.31)	
Alcohol drinking				
Current	1083 (20.41)	630 (17.8)	453 (25.64)	< 0.001***
Ever	281 (5.29)	179 (5.06)	102 (5.77)	
Never	3943 (74.3)	2731 (77.15)	1212 (68.59)	
Regular tea drinking	2231 (42.04)	1413 (39.92)	818 (46.29)	< 0.001***
Regular physical activity	3471 (65.4)	2194 (61.98)	1277 (72.27)	< 0.001***
T2DM (%)				
Yes	809 (15.24)	515 (14.55)	294 (16.64)	< 0.05*
No	4498 (84.76)	3025 (85.45)	1473 (83.36)	
HBP (%)				
Yes	1468 (27.66)	885 (25)	583 (32.99)	< 0.001***
No	3839 (72.34)	2655 (75)	1184 (67.01)	
CKD (%)				
G1 (eGFR ≥ 90)	1597 (30.09)	856 (24.18)	741 (41.94)	< 0.001***
G2 (eGFR < 90)	3710 (69.91)	2684 (75.82)	1026 (58.06)	
FBG, mmol/L	4.9 (4.09, 8.47)	4.9 (4.1, 8.93)	4.93 (4.06, 7.72)	0.207
CREA, μmol/L	78 (48, 109)	71 (46, 103)	86 (55, 119)	< 0.001***
eGFR, mL/min	69.14 (32.54, 129.77)	57.63 (31.85, 122.75)	82.83 (36.69, 139.4)	< 0.001***
SBP, mmHg	127 (103, 166)	125 (102, 164)	130 (107, 168)	< 0.001***
DBP, mmHg	75 (60, 98)	74 (60, 96.5)	78 (61, 100)	< 0.001***
CHOL, mmol/L	4.87 (3.41, 6.74)	4.84 (3.4, 6.77)	4.93 (3.41, 6.71)	0.010**
TG, mmol/L	1.18 (0.51, 3.79)	1.04 (0.47, 3.28)	1.57 (0.66, 4.74)	< 0.001***
LDL‐C, mmol/L	3.13 (1.81, 4.75)	3.08 (1.8, 4.7)	3.24 (1.9, 4.8)	< 0.001***
HDL‐C, mmol/L	1.32 (0.86, 2.15)	1.4 (0.89, 2.23)	1.17 (0.81, 1.84)	< 0.001***
WBC, ×10^9^/L	6.6 (4.5, 9.8)	6.4 (4.4, 9.6)	7 (4.9, 10.1)	< 0.001***
PLT, ×10^9^/L	258 (172, 366)	255 (168.5, 362)	263 (177, 378)	< 0.001***
NLR, ×10^9^/L	1.6 (0.9, 3.13)	1.61 (0.89, 3.17)	1.59 (0.93, 3.07)	0.382
mINFLA score	1 (−6, 9)	0 (−7, 8)	2 (−6, 9)	< 0.001***
TyG index	8.47 (7.53, 9.88)	8.33 (7.46, 9.76)	8.74 (7.78, 10.05)	< 0.001***
UA, μmol/L	358 (216, 543)	314 (203, 405)	463 (375, 606)	< 0.001***

*Note:* Data are median (interquartile range) or number (%).

Abbreviations: BMI, body mass index; CHOL, total cholesterol; CKD, chronic kidney disease; DBP, diastolic blood pressure; DVS, dietary variety score; eGFR, estimated glomerular filtration rate; FBG, fasting blood glucose; HBP, hypertension; HDL‐C, high‐density lipoprotein cholesterol; LDL‐C, low‐density lipoprotein cholesterol; mINFLA score, modified inflammation score; NLR, neutrophil to lymphocyte ratio; PLT, platelet; SBP, systolic blood pressure; T2DM, type 2 diabetes; TG, triglyceride; TyG index, triglyceride–glucose index; UA, serum uric acid; WBC, white blood cell.

* *p* < 0.05.

** *p* < 0.01.

*** *p* < 0.001.

### 3.2 Association of mINFLA Score and TyG Index With HUA Risk

As shown in Table 2, higher mINFLA scores were significantly associated with an increased risk of HUA in the univariable logistic regression model, as well as after adjusting for covariates (model 2: OR = 1.21, 95% CI: 1.11, 1.31, *p* < 0.001). Meanwhile, a significant dose–response relationship was observed, where the risk of HUA increased with ascending mINFLA score by quartiles (*p* for trend < 0.001).

**TABLE 2:** The association of mINFLA score and TyG index with HUA risk.

	No.	Model 1	*p*	Model 2	*p*
		OR (95% CI)		OR (95% CI)	
mINFLA (continuous)		1.35 (1.25, 1.45)	< 0.001***	1.21 (1.11, 1.31)	< 0.001***
mINFLA (quartiles)					
Q1	1646	Ref		Ref	
Q2	1266	1.28 (1.09, 1.51)	< 0.01**	1.10 (0.92, 1.3)	0.300
Q3	1170	1.66 (1.42, 1.95)	< 0.001***	1.39 (1.17, 1.66)	< 0.001***
Q4	1225	1.77 (1.51, 2.08)	< 0.001***	1.42 (1.19, 1.69)	< 0.001***
*p* for trend			< 0.001***		< 0.001***
TyG (continuous)		2.01 (1.85, 2.18)	< 0.001***	1.43 (1.30, 1.57)	< 0.001***
TyG (quartiles)					
Q1	1327	Ref		Ref	
Q2	1327	2.04 (1.69, 2.46)	< 0.001***	1.59 (1.31, 1.94)	< 0.001***
Q3	1327	3.04 (2.53, 3.65)	< 0.001***	1.92 (1.57, 2.35)	< 0.001***
Q4	1326	5.15 (4.3, 6.17)	< 0.001***	2.76 (2.24, 3.39)	< 0.001***
*p* for trend			< 0.001***		< 0.001***

*Note:* mINFLA was converted using the interquartile range (IQR). Model 1: univariable logistic regression model. Model 2: multivariable logistic regression model adjusted for age, BMI, DVS, sleep hours, sex, region, ethnicity, education, annual income, marriage, smoking, alcohol drinking, regular tea drinking and regular physical activity.

Abbreviations: HUA, hyperuricemia; mINFLA score, modified inflammation score; TyG index, triglyceride–glucose index.

** *p* < 0.01.

*** *p* < 0.001.

Similarly, higher TyG index levels were significantly associated with increased HUA risk in both univariable and multivariable analyses (model 2: OR = 1.43, 95% CI: 1.30, 1.57, *p* < 0.001). A significant dose–response relationship was also evident, where HUA risk increased with elevated TyG by quartiles (*p* for trend < 0.001).

### 3.3 Association of mINFLA Score and TyG Index With Serum UA Levels

As shown in Table 3, higher mINFLA scores were significantly associated with an increased level of UA in both univariable and multivariable linear regression models (*β* = 7.55, 95% CI: 4.61, 10.48, *p* < 0.001 in multivariable model). A significant dose–response relationship was observed, where the levels of UA increased with ascending mINFLA score by quartiles (*p* for trend < 0.001). That is, compared to the lowest quartile, participants in the second (*β* = 7.21, 95% CI: 1.29, 13.13), third (*β* = 11.61, 95% CI: 5.5, 17.71), and fourth (*β* = 14.04, 95% CI: 7.94, 20.13) quartiles had progressively higher levels of UA (all *p* < 0.001).

**TABLE 3:** The association of mINFLA score and TyG index with serum UA levels.

		Model 1	*p*	Model 2	*p*
		*β* (95% CI)		*β* (95% CI)	
mINFLA (continuous)		15.98 (12.43, 19.54)	< 0.001***	7.55 (4.61, 10.48)	< 0.001***
mINFLA (quartiles)					
Q1	1646				
Q2	1266	19.22 (11.88, 26.56)	< 0.001***	7.21 (1.29, 13.13)	< 0.05*
Q3	1170	26.42 (18.91, 33.93)	< 0.001***	11.61 (5.5, 17.71)	< 0.001***
Q4	1225	30.98 (23.57, 38.39)	< 0.001***	14.04 (7.94, 20.13)	< 0.001***
*p* for trend			< 0.001***		< 0.001***
TyG (continuous)		39.52 (36.04, 43.01)	< 0.001***	13.46 (10.15, 16.78)	< 0.001***
TyG (quartiles)					
Q1	1327				
Q2	1327	35.03 (27.76, 42.3)	< 0.001***	13.8 (7.54, 20.05)	< 0.001***
Q3	1327	62.13 (54.86, 69.4)	< 0.001***	24.72 (18.17, 31.27)	< 0.001***
Q4	1326	87.8 (80.53, 95.07)	< 0.001***	37.08 (30.12, 44.03)	< 0.001***
*p* for trend			< 0.001***		< 0.001***

*Note:* mINFLA was converted using the interquartile range (IQR). Model 1: univariable linear regression model. Model 2: multivariable linear regression model adjusted for age, BMI, DVS, sleep hours, sex, region, ethnicity, education, annual income, marriage, smoking, alcohol drinking, regular tea drinking and regular physical activity.

Abbreviations: mINFLA score, modified inflammation score; TyG index, triglyceride–glucose index; UA, serum uric acid.

* *p* < 0.05.

*** *p* < 0.001.

Similarly, higher TyG index levels were significantly associated with elevated UA levels in both univariable and multivariable analyses (multivariable model: *β* = 13.46, 95% CI: 10.15, 16.78, *p* < 0.001). A significant dose–response relationship was also evident (*p* for trend < 0.001). That is, when compared to the lowest quartile, the levels of UA were significantly elevated in the second (*β* = 13.8, 95% CI: 7.54, 20.05), third (*β* = 24.72, 95% CI: 18.17, 31.27), and fourth quartiles (*β* = 37.08, 95% CI: 30.12, 44.03) of TyG index (all *p* < 0.001).

### 3.4 Mediation Effect Analysis

**FIGURE 1:**
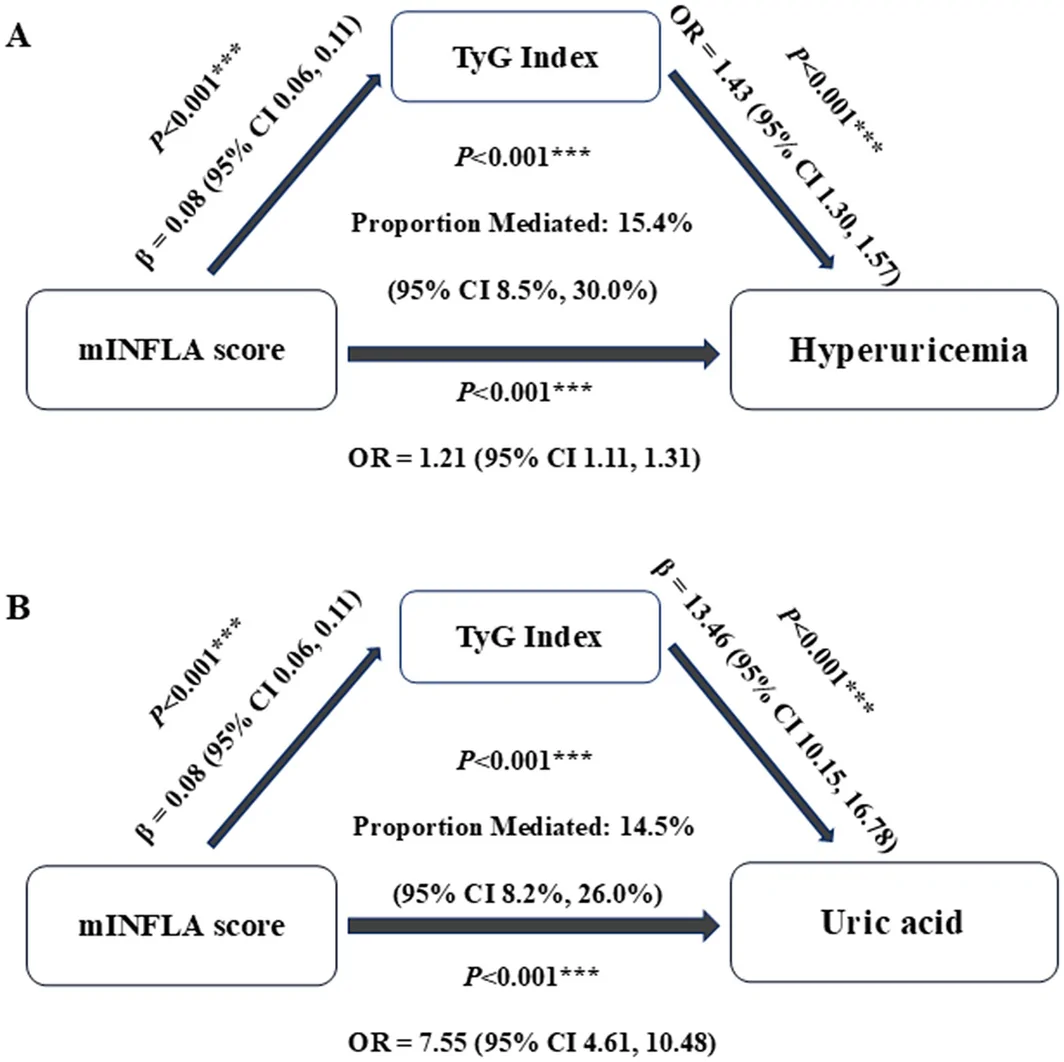
Mediation analysis. (A) Mediation effects of TyG index on the association between mINFLA score with HUA risk. (B) Mediation effects of TyG index on the association between mINFLA score with UA levels. Mediation analysis was conducted using the mediation package in R (version 4.5.1) with 5000 bootstrap samples. All other statistical analyses were performed using Stata (version 12, StataCorp LP, USA). HUA, hyperuricemia; mINFLA score, modified inflammation score; TyG index, triglyceride–glucose index; UA, serum uric acid. *p < 0.05, **p < 0.01, ***p < 0.001.

Figure 1 illustrated the potential mediating roles of the TyG index in the association between the mINFLA score and HUA risk or UA levels. We found that a higher mINFLA score was significantly associated with a higher TyG index (*β* = 0.08, *p* < 0.001). Meanwhile, a higher TyG index was significantly associated with increased HUA risk (OR = 1.43, *p* < 0.001) and UA levels (*β* = 13.46, *p* < 0.001). The mediation proportions in the associations of the mINFLA score with HUA risk and UA levels were 15.4% (95% CI: 8.5%, 30%) and 14.5% (95% CI: 8.2%, 26%), respectively.

### 3.5 Interaction and Combined Effect Analysis

A significant interaction effect of mINFLA score and TyG index on HUA risk was observed in both unadjusted and adjusted models (*p* for interaction = 0.020 and 0.014, respectively). Furthermore, a synergistic joint effect of the two markers was evident. Compared to those with low mINFLA score and low TyG index, participants had a significantly increased hyperuricemia risk by elevated mINFLA score and TyG index (*p* for trend < 0.0001). Particularly, the group of High mINFLA & High TyG had the greatest risk (OR = 2.04, 95% CI: 1.69, 2.47), followed by the High mINFLA & Low TyG group (OR = 1.64, 95% CI: 1.32, 2.03) (Table 4).

**TABLE 4:** The interaction and combined effect of mINFLA score and TyG index on HUA risk.

	*N*	Model 1	*p*	*p* for interaction	Model 2	*p*	*p* for interaction
		OR (95% CI)			OR (95% CI)		
mINFLA*TyG				0.020			0.014
Low mINFLA & low TyG	1202	ref			ref		
Low mINFLA & high TyG	1464	1.28 (1.06, 1.53)	< 0.05*		1.20 (0.99, 1.46)	0.067	
High mINFLA & low TyG	863	2.41 (1.98, 2.93)	< 0.001***		1.64 (1.32, 2.03)	< 0.001***	
High mINFLA & high TyG	1778	3.48 (2.94, 4.12)	< 0.001***		2.04 (1.69, 2.47)	< 0.001***	
*P* for trend			< 0.001***			< 0.001***	

*Note:* Model 1: univariable logistic regression model. Model 2: multivariable logistic regression model adjusted for age, BMI, DVS, sleep hours, sex, region, ethnicity, education, annual income, marriage, smoking, alcohol drinking, regular tea drinking and regular physical activity.

Abbreviations: HUA, hyperuricemia; mINFLA score, modified inflammation score; TyG index, triglyceride–glucose index.

* *p* < 0.05.

*** *p* < 0.001.

However, no interaction effect was found between mINFLA score and TyG index on serum UA levels (*p* for interaction > 0.05) (Table 5). Notably, the joint effect analysis revealed a significant and graded positive association. Compared to the reference group (Low mINFLA & Low TyG), serum UA levels were substantially higher in the combined exposure groups. In the fully adjusted model (Model 2), the greatest increase was observed in the High mINFLA & High TyG group (*β* = 30.13, 95% CI: 23.76, 36.51), followed by the High mINFLA & Low TyG group (*β* = 16.89, 95% CI: 9.61, 24.18). A significant dose–response relationship was observed by the trend test (*p* for trend < 0.0001).

**TABLE 5:** The interaction and combined effects of mINFLA score and TyG index on serum UA levels.

	*N*	Model1	*p*	*p* for interaction	Model2	*p*	*p* for interaction
		*β* (95% CI)			*β* (95% CI)		
mINFLA*TyG				0.462			0.151
Low mINFLA & Low TyG	1202	ref			ref		
Low mINFLA & High TyG	1464	9.27 (1.93, 16.62)	< 0.05*		5.55 (−0.58, 11.67)	0.100	
High mINFLA & Low TyG	863	45.57 (37.14, 53.99)	< 0.001***		16.89 (9.61, 24.18)	< 0.001***	
High mINFLA & High TyG	1778	71.07 (64.02, 78.12)	< 0.001***		30.13 (23.76, 36.51)	< 0.001***	
*P* for trend			< 0.001***			< 0.001***	

*Note:* Model 1: univariable linear regression model. Model 2: multivariable linear regression model adjusted for age, BMI, DVS, sleep hours, sex, region, ethnicity, education, annual income, marriage, smoking, alcohol drinking, regular tea drinking and regular physical activity.

Abbreviations: mINFLA score, modified inflammation score; TyG index, triglyceride–glucose index; UA, serum uric acid.

* *p* < 0.05.

*** *p* < 0.001.

### 3.6 Subgroup Analysis

In subgroup analyses stratified by age, sex, status of T2DM, obesity, CKD and HBP, the joint effects of mINFLA score and TyG index on HUA risk and UA levels remained significant and exhibited a positive dose–response relationship (*p* for trend < 0.05). Participants with both high mINFLA score and high TyG index consistently showed the highest HUA risk and UA levels. Significant multiplicative interactions were observed for sex, T2DM and HBP with respect to both HUA risk and UA levels (*p* for interaction < 0.05, Tables S1–S2).

## 4 Discussion

In this cross‐sectional study of 5307 participants from Southern China, we found positive dose‐dependent associations of both the TyG index and mINFLA score with HUA risk and UA levels, where mINFLA score is a composite marker of low‐grade inflammation and TyG index serves as a reliable alternative for IR. Meanwhile, a significant multiplicative interaction and joint effect of the two indices on HUA risk and UA levels were also identified, with the highest risk observed among individuals exhibiting concurrent elevations in both markers. Notably, the TyG index significantly mediated 15.4% of the association between mINFLA score and HUA risk, and 14.5% of its association with UA levels. Collectively, these findings suggest that systemic inflammation and IR are not only independent risk factors for HUA but also interact and jointly contribute to its development, with IR partially mediating the inflammatory pathway in uric acid dysregulation.

Our findings on the independent association between systemic inflammation and HUA risk are firmly supported by established biological mechanisms. Uric acid homeostasis is critically regulated by urate transporters, including organic anion transporter 1 (OAT1), ATP‐binding cassette subfamily G member 2 (ABCG2), renal urate transporter 1 (URAT1), and glucose transporter 9 (GLUT9) [28]. Improving the inflammatory state (e.g., IL‐1β, IL‐6β and TNF‐α) to repair damage in kidney will help restore uric acid balance, mainly achieved by enhancing the expression of excretory transporters like OAT1 and ABCG2 while reducing reabsorptive transporters such as URAT1 and GLUT9 [29, 30]. This is corroborated by several population studies showing that systemic inflammation [31, 32] could serve as an important mediating factors on HUA, with elevated levels of pro‐inflammatory cytokines [3, 33, 34] and composite hematologic indices [35, 36] in hyperuricemic individuals. The mINFLA score, integrating WBC, PLT, and NLR [18], likely captures this persistent low‐grade inflammatory state more comprehensively than single markers, explaining its robust association with HUA risk in our study.

Similarly, the independent link between the TyG index (IR) and HUA is well‐grounded in pathophysiology. IR, a hallmark of HUA, stimulates renal uric acid reabsorption, primarily by enhancing the activity of the basolateral urate transporter GLUT9 in the renal proximal tubule, thereby reducing uric acid excretion, while IR related Insulin‐Like Growth Factor‐1 (IGF‐1) can reduce serum UA by stimulating OAT1 and ABCG2 and inhibiting insulin's action [37]. This mechanism pathway is confirmed by the negative correlations between IR and uric acid clearance as well as fractional excretion of urate (FEUA) [4, 38]. While some debate exists regarding the direction of causality [39], Mendelian randomization studies largely support a unidirectional causal pathway from IR to HUA [5].

The novelty of this study lies in the integration of these two pathways. We demonstrated a significant positive interaction between mINFLA score and TyG index, suggesting that their combined effect on HUA risk exceeds the sum of their individual contributions. Furthermore, we identified the TyG index as a significant mediating factor in the inflammation‐HUA pathway. This finding is supported by the established biology, as chronic inflammation is a well‐recognised driver of IR.

In obesity, dysfunctional adipose tissue secretes pro‐inflammatory adipokines (e.g., TNF‐α, leptin, resistin) while reducing anti‐inflammatory adipokines. These cytokines disrupt insulin signalling via multiple mechanisms. For instance, TNF‐α activates JNK and IKKβ/NF‐κB pathways, impairing insulin receptor substrate phosphorylation; leptin and resistin reduce AMPK activity and GLUT4 translocation, altering insulin‐sensitive gene expression. These signals also exacerbate inflammation through TLR4/IL‐6 cascades, creating a vicious cycle that aggravates insulin resistance [40, 41]. Meanwhile, reduced adiponectin diminishes PPARγ activity, further compromising insulin sensitivity [42]. Macrophage infiltration, triggered by factors such as IgG accumulation, sustains adipose tissue inflammation and further impairs insulin signalling [43, 44].

The resultant hyperinsulinemia directly stimulates uric acid reabsorption, predominantly by upregulating GLUT9a and URAT1 via AKT‐mediated pathways in the proximal renal tubules [37, 38]. Thus, systemic inflammation drives hyperuricemia through a coherent pathway: inflammatory cytokines induce insulin resistance and hyperinsulinemia, which in turn enhances renal uric acid reabsorption, elevating serum uric acid levels. This mechanistic cascade is corroborated by intervention studies demonstrating that anti‐inflammatory agents (e.g., AMPK activators, NF‐κB inhibitors) concurrently improve insulin sensitivity [45, 46, 47, 48], and that SGLT2 inhibitors reduce both inflammatory markers (e.g., IL‐6), insulin levels and UA levels [49].

The “inflammation → insulin resistance → HUA” pathway observed in the southern Chinese population is consistent with the core pathophysiology found elsewhere. Our finding that insulin resistance is a risk factor for hyperuricemia aligns with evidence from both northern Chinese [9] and European cohorts [5]. However, a systematic review revealed marked regional disparities in hyperuricemia prevalence across mainland China, with the southern region exhibiting the highest prevalence (25.5%) [2]. This disparity likely reflects differences in dietary staples (rice vs. wheat) and seafood intake [50, 51], alongside genetic predisposition [52].

The mediation effect in this study is partial, indicating that the TyG index explains only a portion of the association between mINFLA score and hyperuricemia. This is biologically more plausible than complete mediation and implies the existence of other concurrent pathways. For instance, inflammation may further influence hyperuricemia via obesity‐related mechanisms, as supported by our additional mediation analysis showing that BMI accounted for a substantial proportion of the effect (proportion mediated = 44.2%; 95% CI: 34.0%, 60.5%; *p* < 0.001; Figure S4). Another potential pathway is through direct or indirect renal function impairment. Chronic inflammation can reduce the glomerular filtration rate (eGFR), thereby reducing uric acid filtration and promoting hyperuricemia. Our analysis also indicated a modest but significant mediating role of eGFR (proportion mediated = 5.5%; 95% CI: 2%, 12%; *p* < 0.001; Figure S5). Further studies are warranted to elucidate the network of effects linking inflammation to hyperuricemia.

Consistently, we also found the interaction and joint effects of mINFLA score and TyG index on HUA risk stratified by sex, hypertension and T2DM status, which strengthens the generalizability of our core findings. From a clinical and public health perspective, our results underscore the importance of a holistic approach to HUA prevention and management. Assessing both inflammatory status (e.g., via mINFLA score) and insulin resistance (e.g., via TyG index) could improve risk stratification. Therapeutic strategies should consider concurrently addressing both conditions, potentially through anti‐inflammatory diets (e.g., Mediterranean diet [53] and tea [54]), or pharmacological agents (e.g., metformin [55] and Si‐Miao‐San [56]) that target both pathways.

Our study has several strengths, including the large sample size, the use of a novel accessible composite inflammatory index, and the comprehensive analysis of independent, interaction, joint, and mediation effects of mINFLA score and TyG index on HUA risk. Particularly, our findings identified the TyG index as a mediator of the inflammatory index on the risk of HUA and further uncover the significant interaction and synergistic effect between inflammation and insulin resistance on HUA. However, the limitations need to be considered. Firstly, the cross‐sectional study design inherently limits causal interpretation of the independent associations that we demonstrated between mINFLA score, TyG index and HUA risk. Although we adjusted for numerous potential confounders, residual confounding cannot be entirely ruled out. Secondly, the mediation analysis showed that the confidence intervals for some of the estimated mediation effects were relatively wide (e.g., 15.4% with 95% CI 8.5%–30%; 14.5% with 95% CI 8.2%–26%). Such wide intervals are not uncommon in complex mediation models based on bootstrap methods [57, 58], which may be influenced by sample size and intricate interrelationships among variables. Although the width of the intervals limits the precision with which the exact magnitude of the mediation effect can be determined, it is noteworthy that the lower bounds of all intervals remain substantially above zero. This supports the statistical significance of the mediating role of the TyG index in the association between inflammation and hyperuricemia. The point estimates (e.g., 15.4%) should therefore be interpreted as preliminary references, and their precise contribution warrants further verification in future studies with larger sample sizes. Thirdly, while a significant interaction was observed on the risk of HUA, no such interaction was found for continuous uric acid levels. This discrepancy may suggest a “threshold effect” characteristic, where the synergistic effect predominantly affecting the transition from normal to hyperuricemic status rather than determining uric acid concentration per se. This highlights the different pathophysiological or clinical implications that may be captured by analysing a disease status compared to its underlying biomarker. Finally, our study population was drawn from a single province in Southern China, which may limit the generalizability of our findings to other ethnic or geographic populations.

## 5 Conclusions

In conclusion, both TyG index and mINFLA score were independent risk factors for hyperuricemia (HUA) in this Southern Chinese population. These two factors exhibit a significant interaction and joint effect on HUA risk. Moreover, the TyG index acts as a partial mediator in the pathway linking systemic inflammation to HUA risk. These findings suggest that integrated assessment and simultaneous management of both inflammatory status and insulin resistance may be crucial for the effective prevention and control of HUA. Future prospective cohort studies and mechanistic investigations are warranted to confirm these associations and elucidate the underlying causal pathway.

## Author Contributions

Conceptualization: R.L. and H.Z. Data curation: X.H., P.C. and Y.G. Formal analysis: X.H., P.C. and Y.G. Funding acquisition: R.L. Investigation: X.H., P.C. and Y.G. Methodology: X.H., P.C. and Y.G. Project administration: Z.M. Resources: Z.M., R.L. and H.Z. Software: X.H., P.C. and Y.G. Supervision: R.L. and H.Z. Validation: Z.M., R.L. and H.Z. Visualisation: Y.G. Writing – original draft: X.H. and P.C. Writing – review and editing: R.L. and H.Z.

## Funding

This work was supported by the Natural Science Foundation of Guangxi Province (No. 2025GXNSFDA069012, 2018GXNSFDA050019), and the National Natural Science Foundation of China (No. 82273618, 82060593).

## Consent

All authors have approved the manuscript for submission.

## Conflicts of Interest

The authors declare no conflicts of interest.

## Supporting information


**Figure S1:** Flow chart of participants selection. A total of 6368 participants were initially recruited, and 5307 were included in the final analysis after exclusions.

**Figure S2:** Association between the mINFLA score and hyperuricemia (HUA) risk. The odds ratios (ORs) and 95% confidence intervals (CIs) were derived from restricted cubic spline (RCS) regression, adjusted for age, body mass index (BMI), dietary variety score (DVS), sleep hours, sex, region, ethnicity, education, annual income, marital status, smoking, alcohol consumption, regular tea drinking, and regular physical activity. The solid line represents the adjusted ORs fitted by the RCS model, and the grey area represents the 95% CI. *P for overall* association < 0.05 indicates a statistically significant association; *P for nonlinear* < 0.05 suggests a significant non‐linear relationship. BMI, body mass index; DVS, dietary variety score; HUA, hyperuricemia; mINFLA score, modified inflammation score; RCS, restricted cubic splines.

**Figure S3:** Association between the TyG index and hyperuricemia (HUA) risk. The odds ratios (ORs) and 95% confidence intervals (CIs) were derived from restricted cubic spline (RCS) regression, adjusted for age, body mass index (BMI), dietary variety score (DVS), sleep hours, sex, region, ethnicity, education, annual income, marital status, smoking, alcohol consumption, regular tea drinking, and regular physical activity. The solid line represents the adjusted ORs fitted by the RCS model, and the grey area represents the 95% CI. *P for overall* association < 0.05 indicates a statistically significant association; *P for nonlinear* < 0.05 suggests a significant non‐linear relationship. BMI, body mass index; DVS, dietary variety score; HUA, hyperuricemia; RCS, restricted cubic splines; TyG index, triglyceride–glucose index.

**Figure S4:** Mediation analysis. (A) Mediation effect of BMI on the association between the mINFLA score and HUA risk. (B) Mediation effect of BMI on the association between the mINFLA score and UA levels. Mediation analysis was conducted using the mediation package in R (version 4.5.1) with 5000 bootstrap samples. All other statistical analyses were performed using Stata (version 12, StataCorp LP, USA). BMI, body mass index; HUA, hyperuricemia; mINFLA score, modified inflammation score; UA, serum uric acid. *p < 0.05, **p < 0.01, ***p < 0.001.

**Figure S5:** Mediation analysis. (A) Mediation effect of eGFR on the association between the mINFLA score and HUA risk. (B) Mediation effect of eGFR on the association between the mINFLA score and UA levels. Mediation analysis was conducted using the mediation package in R (version 4.5.1) with 5000 bootstrap samples. All other statistical analyses were performed using Stata (version 12, StataCorp LP, USA). eGFR, estimated glomerular filtration rate; HUA, hyperuricemia; mINFLA score, modified inflammation score; UA, serum uric acid. *p < 0.05, **p < 0.01, ***p < 0.001.

**Table S1:** Subgroup analysis for the combined effect of mINFLA score and TyG index on HUA risk.
**Table S2:** Subgroup analysis for the combined effect of mINFLA score and TyG index on serum UA levels.

## Data Availability

The data of this study can be requested from the corresponding author's email as needed.
