# Supplementary material for: Interaction and Joint Effects of Inflammation and Triglyceride–Glucose (TyG) Index on Hyperuricemia Risk and the Mediating Role of TyG: A Cross‐Sectional Study
Source: Endocrinol Diabetes Metab. 2026 Mar 30;9(3):e70205. doi: 10.1002/edm2.70205 (PMC13580815; doi:10.1002/edm2.70205)
Supplement: Supplementary file 1 [file EDM2-9-e70205-s004.docx]

**Table S1 Subgroup analysis for the combined effect of mINFLA score and TyG index on HUA risk**

| Subgroups | Low mINFLA  & High TyG | High mINFLA  & Low TyG | High mINFLA  & High TyG | *P* for trend | *P* for interaction |
| --- | --- | --- | --- | --- | --- |
| Age, years |  |  |  |  | 0.517 |
| <40 (1994/5307) | 1.33 (0.99, 1.78) | 1.50 (1.00, 2.25) ^*^ | 2.05 (1.49, 2.81) ^***^ | <0.001^***^ |  |
| 40-60 (1741/5307) | 1.08 (0.73, 1.58) | 1.73 (1.19, 2.53) ^**^ | 1.87 (1.33, 2.63) ^***^ | <0.001^***^ |  |
| ≥60 (1572/5307) | 1.13 (0.76, 1.67) | 1.5 (1.03, 2.19) ^*^ | 1.93 (1.36, 2.74) ^***^ | <0.001^***^ |  |
| Sex |  |  |  |  | <0.001^***^ |
| Male (3423/5307) | 1.19 (0.95, 1.51) | 1.56 (1.22, 1.99) ^***^ | 1.79 (1.44, 2.22) ^***^ | <0.001^***^ |  |
| Female (1884/5307) | 1.26 (0.85, 1.85) | 1.64 (1.03, 2.61) ^*^ | 2.55 (1.71, 3.8) ^***^ | <0.001^***^ |  |
| T2DM |  |  |  |  | <0.05^*^ |
| Yes (809/5307) | 1.76 (0.78, 3.99) | 1.48 (0.72, 3.07) | 1.70 (0.85, 3.42) | 0.239 |  |
| No (4498/5307) | 1.14 (0.93, 1.40) | 1.69 (1.34, 2.14) ^***^ | 2.17 (1.77, 2.66) ^***^ | <0.001^***^ |  |
| Obesity |  |  |  |  | 0.076 |
| Yes (465/5307) | 0.58 (0.22, 1.55) | 0.82 (0.33, 2.08) | 1.05 (0.44, 2.49) | 0.150 |  |
| No (4842/5307) | 1.31 (1.07, 1.59) ^**^ | 2.04 (1.64, 2.54) ^***^ | 2.74 (2.27, 3.31) ^***^ | <0.001^***^ |  |
| CKD |  |  |  |  | 0.386 |
| Yes (1597/5307) | 1.21 (0.85, 1.73) | 1.57 (1.07, 2.29) ^*^ | 1.89 (1.35, 2.63) ^***^ | <0.001^***^ |  |
| No (3710/5307) | 1.18 (0.93, 1.49) | 1.72 (1.32, 2.24) ^***^ | 2.08 (1.65, 2.62) ^***^ | <0.001^***^ |  |
| HBP |  |  |  |  | <0.05^*^ |
| Yes (1468/5307) | 1.17 (0.77, 1.78) | 1.29 (0.85, 1.96) | 1.59 (1.09, 2.32) ^*^ | <0.01^**^ |  |
| No (3839/5307) | 1.17 (0.94, 1.46) | 1.78 (1.38, 2.31) ^***^ | 2.15 (1.72, 2.68) ^***^ | <0.001^***^ |  |

The multivariable logistic regression model was adjusted for age, BMI, DVS, sleep hours, sex, region, ethnicity, education, annual income, marriage, smoking, alcohol drinking, regular tea drinking and regular physical activity, obesity, HBP, and CKD. Superscript letters indicate significant differences from the reference group (Low mINFLA & Low TyG).

*P*-value: ^*^*P* < 0.05, ^**^*P* < 0.01, ^***^*P* < 0.001.

Abbreviation: mINFLA score, modified inflammation score. TyG index, triglyceride–glucose index. HUA: hyperuricemia. T2DM, type 2 diabetes. HBP, hypertension. CKD, chronic kidney disease. BMI, body mass index. DVS, dietary variety score.

**Table S2 Subgroup analysis for the combined effect of mINFLA score and TyG index on serum UA levels**

| Subgroups | Low mINFLA  & High TyG | High mINFLA  & Low TyG | High mINFLA  & High TyG | *P* for trend | *P* for interaction |
| --- | --- | --- | --- | --- | --- |
| Age, years |  |  |  |  |  |
| <40 (1994/5307) | 4.21 (-4.09, 12.5) | 11.08 (-2, 24.15) | 26.47 (16.31, 36.63) ^***^ | <0.001^***^ | 0.368 |
| 40-60 (1741/5307) | 3.81 (-8.12, 15.74) | 23.41 (10.79, 36.03) ^***^ | 28.63 (17.31, 39.95) ^***^ | <0.001^***^ |  |
| ≥60 (1572/5307) | 7.73 (-4.98, 20.44) | 13.09 (0.14, 26.04) ^*^ | 30 (17.93, 42.08) ^***^ | <0.001^***^ |  |
| Sex |  |  |  |  | <0.05^*^ |
| Male (3423/5307) | 8.15 (-0.8, 17.1) | 15.16 (5.4, 24.91) ^**^ | 27.23 (18.67, 35.78) ^***^ | <0.001^***^ |  |
| Female (1884/5307) | 2.9 (-4.53, 10.33) | 16.76 (6.39, 27.13) ^**^ | 30.84 (21.65, 40.04) ^***^ | <0.001^***^ |  |
| T2DM |  |  |  |  | <0.05^*^ |
| Yes (809/5307) | 33.91 (0.79, 67.03) ^*^ | 9.36 (-19.26, 37.99) | 25.63 (-1.64, 52.90) | 0.156 |  |
| No (4,498/5307) | 3.41 (-2.56, 9.38) | 21.61 (14.09, 29.13) ^***^ | 34.21 (27.67, 40.74) ^***^ | <0.001^***^ |  |
| Obesity |  |  |  |  |  |
| Yes (465/5307) | -36.78 (-81.96, 8.39) | -16.72 (-59.58, 26.13) | 4.14 (-35.79, 44.07) | <0.05^*^ | 0.438 |
| No (4842/5307) | 9.83 (3.63, 16.04) ^**^ | 27.65 (20.19, 35.11) ^***^ | 43.68 (37.33, 50.03) ^***^ | <0.001^***^ |  |
| CKD |  |  |  |  | 0.561 |
| Yes (1597/5307) | 9.07 (-5, 23.15) | 17.83 (2.64, 33.02) ^*^ | 32.76 (19.54, 45.99) ^***^ | <0.001^***^ |  |
| No (3710/5307) | 4.43 (-2.21, 11.06) | 18.88 (10.65, 27.12) ^***^ | 28.32 (21.08, 35.57) ^***^ | <0.001^***^ |  |
| HBP |  |  |  |  | <0.05^*^ |
| Yes (1468/5307） | 8.51 (-7.02, 24.03) | 13.06 (-2.77, 28.89) | 22.24 (7.8, 36.68) ^**^ | <0.01^**^ |  |
| No (3839/5307) | 3.59 (-2.92, 10.09) | 18.64 (10.39, 26.88) ^***^ | 32.61 (25.5, 39.72) ^***^ | <0.001^***^ |  |

The multivariable logistic regression model was adjusted for age, BMI, DVS, sleep hours, sex, region, ethnicity, education, annual income, marriage, smoking, alcohol drinking, regular tea drinking and regular physical activity, obesity, HBP, and CKD. Superscript letters indicate significant differences from the reference group (Low mINFLA & Low TyG).

*P*-value: ^*^*P* < 0.05, ^**^*P* < 0.01, ^***^*P* < 0.001.

Abbreviation: mINFLA score, modified inflammation score. TyG index, triglyceride–glucose index. UA, serum uric acid. T2DM, type 2 diabetes. HBP, hypertension. CKD, chronic kidney disease. BMI, body mass index. DVS, dietary variety score.
